# Isolated pectoralis minor tear at the costal origin: a case-based review of the literature

**DOI:** 10.1186/s12891-025-09454-6

**Published:** 2026-01-26

**Authors:** Nezih Ziroglu, Ali Can Koluman

**Affiliations:** 1 Department of Orthopedic and Traumatology, Acibadem University Atakent Hospital, Istanbul, Türkiye 34638 Turkey; 2 Department of Orthopedic Prosthetics and Orthotics, Vocational School of Health Services, Acibadem Mehmet Ali Aydınlar University, Istanbul, 34638 Türkiye; 3 Department of Orthopedic and Traumatology, Bakirkoy Dr. Sadi Konuk Training and Research Hospital, Istanbul, 34147 Turkey

**Keywords:** Pectoralis minor, Muscle tear, Costal origin, MRI, Anterior chest wall pain, Scapular stabilizers, Case report

## Abstract

**Background:**

Isolated tears of the pectoralis minor (PMi) are rare and often misdiagnosed as pectoralis major or costochondral injuries. Since the first report in 2004, only eleven cases have been described in the English-language literature, all involving the myotendinous junction or coracoid insertion. A tear at the costal origin has not previously been reported.

**Case presentation:**

A 46-year-old male developed acute anterior chest pain after pulling a heavy hospital bed. Physical examination revealed focal tenderness over the third to fifth ribs and pain with resisted internal rotation. Magnetic resonance imaging demonstrated a partial tear of the pectoralis minor at its costal origin with associated rib bone marrow edema, while the coracoid insertion and pectoralis major were intact. The patient was treated nonoperatively with analgesics and a structured physiotherapy program emphasizing scapular stabilization, resulting in complete symptom resolution within six weeks.

**Literature review:**

A structured search of PubMed and Scopus identified eleven previously reported cases of isolated PMi tear published between 2004 and 2021. Most injuries occurred in contact or overhead athletes and involved the myotendinous or insertional region. All cases were managed conservatively with favorable clinical outcomes.

**Conclusion:**

This case represents the first documented costal origin tear of the pectoralis minor, associated with rib bone marrow edema following a low-energy, non-sporting mechanism. The findings expand the anatomical spectrum of PMi injuries and highlight the importance of considering this diagnosis in patients with anterior chest wall pain and an intact pectoralis major. MRI is essential for accurate localization of the lesion and exclusion of concomitant injury. Conservative management remains an effective treatment strategy.

**Supplementary Information:**

The online version contains supplementary material available at 10.1186/s12891-025-09454-6.

## Introduction

Tears of the pectoralis major are well recognized, particularly among athletes involved in contact or resistance-based sports. In contrast, isolated tears of the pectoralis minor (PMi) are rare and frequently overlooked because of the muscle’s deep location beneath the pectoralis major and its limited contribution to gross shoulder motion. As a result, PMi injuries are often misdiagnosed as pectoralis major strains, costochondral injuries, or nonspecific anterior chest wall pain [1–3]. 

The pectoralis minor originates from the third to fifth ribs near the costochondral junction and inserts onto the medial border of the coracoid process. Its primary function is scapular stabilization, drawing the scapula anteriorly and inferiorly along the thoracic wall, while it also assists rib elevation during deep inspiration [4, 5]. These biomechanical roles expose the muscle to eccentric traction forces, particularly during sudden scapular retraction or resisted upper extremity loading.

Since the first isolated PMi tear described by Mehallo in 2004 [3], eleven cases have been reported in the English-language literature [1–3, 6–11]. Most injuries occurred in contact or overhead athletes following direct impact or forced abduction–external rotation. Reported tear locations have been limited to the myotendinous junction or the coracoid insertion, and all patients were successfully treated nonoperatively, achieving full functional recovery within 2–12 weeks [1–3, 6–11]. 

Two narrative reviews published in 2019 by Vance et al. and Genel and Kieser further confirmed that all previously reported PMi tears were sports related and confined to the myotendinous or insertional regions [6, 11]. To date, a tear involving the costal origin of the pectoralis minor has not been described.

The present report describes the first documented costal origin tear of the pectoralis minor, associated with rib bone marrow edema following a low-energy, non-sporting mechanism, and provides an updated review of all reported cases.

## Case presentation

A 46-year-old right-hand-dominant male school teacher, who regularly performed recreational pull-up exercises but was otherwise healthy, presented with acute-onset pain in the left anterior chest wall after attempting to pull a heavy hospital bed base. He described a sudden, sharp pain localized to the left pectoral region, without radiation, paresthesia, or an audible pop at the time of injury. The pain was predominantly activity related and was mildly exacerbated at the end of deep inspiration.

He denied any prior trauma, recent infection, corticosteroid use, or underlying systemic disease. There was no history of similar symptoms. Written informed consent was obtained from the patient for publication of this case report and the accompanying images.

### Physical examination

Inspection revealed no visible ecchymosis, deformity, swelling, or contour asymmetry of the anterior chest wall. Palpation elicited marked focal tenderness over the third to fifth ribs near the costochondral junction and adjacent intercostal regions, while costovertebral angle tenderness was absent. Mild tenderness was noted over the coracoid process.

Active and passive shoulder range of motion was nearly full. Internal rotation produced mild discomfort, whereas external rotation was painless and symmetric. Strength testing demonstrated pain during resisted internal rotation and a painful lift-off maneuver without objective weakness; subsequent MRI confirmed an intact subscapularis tendon. Belly-press and bear-hug tests were pain-free, and the contour of the pectoralis major muscle was symmetric and intact.

**Fig. 1:**
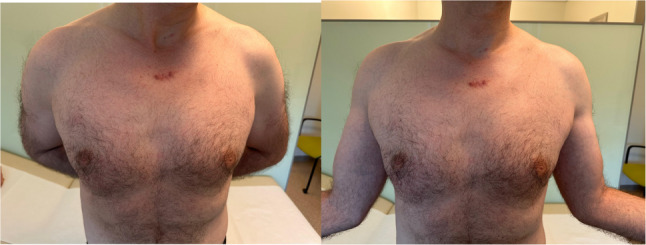
Clinical photographs of the anterior chest wall demonstrating preserved pectoralis major muscle contour without visible ecchymosis or deformity

Overall, the combination of focal costal tenderness, preserved pectoralis major contour, and pain provoked by deep, sub-pectoral maneuvers suggested a deeper anterior chest wall lesion consistent with pectoralis minor injury (Fig. 1).

### Imaging studies

Magnetic resonance imaging (MRI) of the left shoulder demonstrated a partial tear of the pectoralis minor muscle at its costal origin, involving the third and fourth costochondral junctions. Associated bone marrow edema was present in the adjacent ribs, consistent with a traction-related injury mechanism. The coracoid insertion of the pectoralis minor and the pectoralis major muscle were intact.

**Fig. 2:**
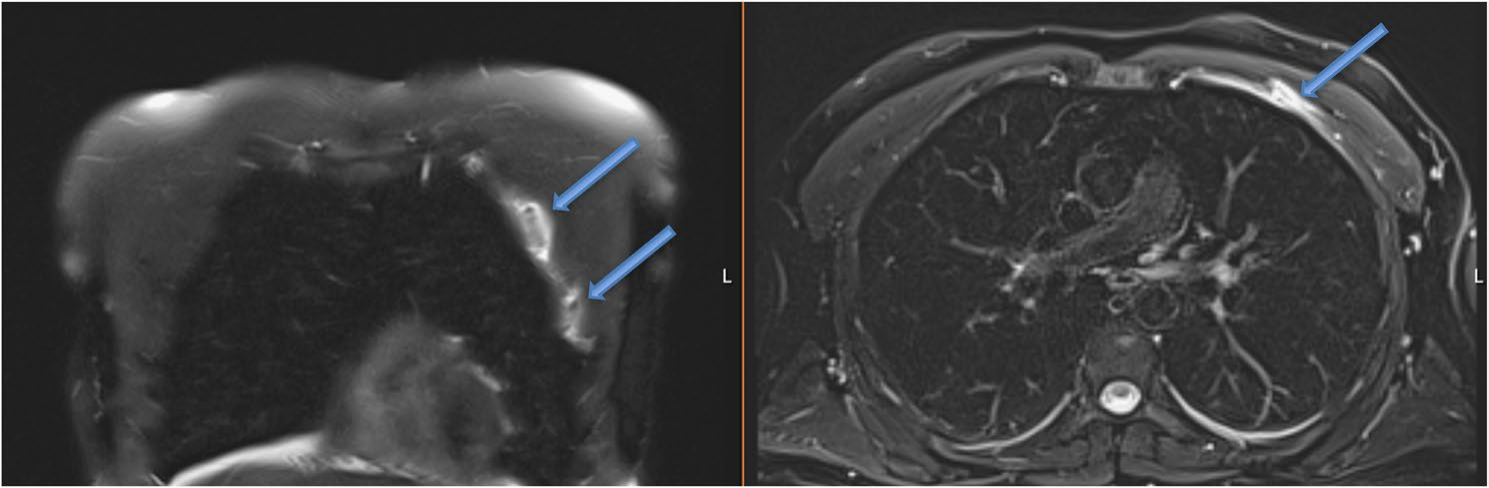
Coronal STIR demonstrates adjacent rib edema. Axial PD-FS MRI shows a partial pectoralis minor tear at the costal origin (arrows) near the 3rd–4th costochondral junction

Additional findings included mild subacromial impingement and low-grade signal changes of the long head of the biceps tendon, without evidence of rotator cuff tear or labral pathology (Fig. 2).

### Management and outcome

The patient was managed nonoperatively with short-term oral analgesics and a structured, progressive physiotherapy program. Shoulder immobilization was intentionally avoided to minimize the risk of stiffness.

During the initial phase (weeks 0–2), activities provoking anterior chest wall pain were restricted. Overhead loading, resisted internal rotation, and forceful scapular retraction were avoided. Gentle, pain-free shoulder range-of-motion exercises were permitted, along with postural correction and breathing control exercises.

In the intermediate phase (weeks 2–4), active range-of-motion exercises were progressively advanced. Scapular stabilization exercises focusing on the serratus anterior and lower trapezius were initiated, while direct stretching or strengthening of the pectoralis minor was deferred until pain resolution.

During the final phase (weeks 4–6), gradual strengthening exercises and functional activities were introduced, including controlled scapular retraction and return-to-activity drills as tolerated.

At six weeks, the patient reported complete resolution of pain with full restoration of shoulder range of motion and strength, allowing return to work and recreational activities without limitation. At the three-month follow-up, he remained asymptomatic with symmetric strength and range of motion, and no evidence of recurrent pain or functional deficit.

### Review of the literature

#### Literature search strategy

A structured literature search was conducted in the PubMed and Scopus databases (last search: October 6, 2025) using the following Boolean search strategy: (“pectoralis minor” AND (“tear” OR “rupture” OR “injury”)). Reference lists of all eligible articles were manually screened to identify additional relevant cases. Studies were included if they reported isolated pectoralis minor tears confirmed by magnetic resonance imaging or intraoperative findings. Reports describing combined pectoralis major injuries or nonspecific anterior chest wall conditions were excluded.

The search yielded 198 records, of which nine primary articles and two review articles met the inclusion criteria. Each review article contained one additional unique case, resulting in a total of eleven previously reported patients with isolated pectoralis minor tear published between 2004 and 2021 [6, 11]. 

**Table 1:** Summary of previously reported cases of isolated pectoralis minor tear (2004–2021) *Data source: pubmed and scopus (search date: October 6*,* 2025). Only isolated PMi tears with MRI confirmation included*

Year	Author (Ref)	Age/Sex	Mechanism of Injury	Level of Tear	Imaging Findings	Management	Clinical Outcome
2004	Mehallo[12]	40 / F	Direct impact (football)	Myotendinous	MRI: edema, P.Major intact	Conservative	Pain-free at 2 wks
2009	Zvijac et al.[3]*	Late 20s / Pro football players (*n* = 2)	Blocking / contact	Myotendinous	MRI-confirmed MTJ tear	Conservative	Return 3–4 wks
2010	Kalra & Neri[2]	25 / M/ Pro hockey player	Collision	Insertional (coracoid)	Tendon retraction at coracoid	Conservative	Full in 4 wks
2012	Li et al.[1]	17 / M/ HS football	Tackle / FOOSH	Insertional	Partial tear at coracoid	Conservative	Recovery by 12 wks
2016	Örücü et al.[8]	HD patient	Spontaneous	Myotendinous	Localized edema	Conservative	Asymptomatic
2018	Colazo et al.[10]	24 / M	Motor vehicle collision	Insertional (partial)	Focal signal near coracoid	Conservative	Full recovery
2018	McNeilan et al.[9]	27 / M/ NFL quarterback	Fall on outstretched arm	Myotendinous	MTJ edema	Conservative	Return 1 wk
2019	Vance et al.[11]	24 / F	Non-contact side-plank	Myotendinous	MTJ edema	Conservative	Full in 6 wks
2019	Genel & Kieser[6]	21 / M	Rugby tackle	Myotendinous	MRI: MTJ tear; P.Major intact	Conservative	Returned to play at 4 wks
2021	Loske et al.[7]	30 / M	Occupational overuse	Insertional	Tear + subclavian vein thrombosis	Conservative + anticoag.	Mild venous sequelae
2025	Present case	46 / M	Low-energy pulling (non-sport)	**Costal origin tear**	Partial tear at 3rd–4th costochondral junction + rib marrow edema	Conservative	Full in 6 wks

*Zvijac et al. reported two athletes; total previously reported cases = 11. Including the present case, total = 12 [3]

Across reported cases, most injuries occurred in young athletes during contact or eccentric-loading activities. Tear location predominantly involved the myotendinous junction [3, 8, 9, 11, 12] or the coracoid insertion [1, 2, 7, 10]. Diagnostic delay was frequently noted, likely related to the deep anatomical position of the pectoralis minor beneath the pectoralis major. In all cases, MRI was essential for confirming tear location and excluding concomitant pectoralis major injury [1–3, 6, 7, 9, 11, 12]. Management was uniformly nonoperative, with return to full activity or sport typically achieved within 2–12 weeks (Table 1). Vascular involvement was reported in a single case, presenting as subclavian vein thrombosis, which also responded to conservative management combined with anticoagulation therapy [7]

##### Position of the present case 

Unlike previously reported cases limited to myotendinous or insertional involvement, the present patient exhibited a tear at the costal origin of the pectoralis minor, accompanied by adjacent rib bone marrow edema following a low-energy, non-sporting mechanism. This finding broadens the recognized anatomical spectrum of pectoralis minor tears and underscores the potential for diagnostic uncertainty when anterior chest wall pain is localized near the costochondral junction rather than the coracoid process.

## Discussion

Isolated tears of the PMi represent an uncommon clinical entity and are frequently overshadowed by injuries of the pectoralis major or rotator cuff. Owing to its deep anatomical location beneath the pectoralis major and its close relationship to the coracoid process, clinical diagnosis is often challenging and delayed.

To date, eleven cases of isolated PMi tear have been reported in the English-language literature, all involving either the myotendinous junction or the coracoid insertion. The present report represents the twelfth documented case overall and the first involving the costal origin, thereby expanding the recognized anatomical and clinical spectrum of this rare injury.

### Mechanism and anatomical considerations

The PMi functions as a dynamic stabilizer of the scapula, drawing it anteriorly and inferiorly during protraction and forward elevation [4, 5]. Sudden or excessive traction, particularly when the scapula is fixed against the thoracic wall, may generate eccentric loading across the muscle’s attachment sites.

In previously reported cases, such traction most commonly occurred during blocking, tackling, or forced abduction–external rotation in contact or overhead sports [1–3, 9–12]. The case described by Genel and Kieser [6], involving a rugby player with a myotendinous tear sustained during a tackle, exemplifies this high-load injury mechanism.

In contrast, the present patient sustained injury during a low-energy, non-sporting pulling maneuver while attempting to move a hospital bed. This observation suggests that even submaximal contraction may result in PMi injury when the rib cage is fixed and the scapula retracts abruptly, generating tensile overload at the costal origin. The tendon–bone interface near the costochondral junction may therefore represent a vulnerable site under such conditions. The presence of adjacent rib bone marrow edema on MRI further supports a traction-related injury mechanism at the proximal attachment.

From an anatomical perspective, PMi tears can now be categorized into three distinct levels:

1. Insertional (coracoid process) – reported by Kalra [2], Li [1], and Loske [7];
2. Myotendinous junction – reported by Mehallo [12], Zvijac [3], Örücü [8], McNeilan [9], Vance [11], and Genel & Kieser [6];
3. Costal origin (costal attachment) – the current case (first documented).

**Fig. 3:**
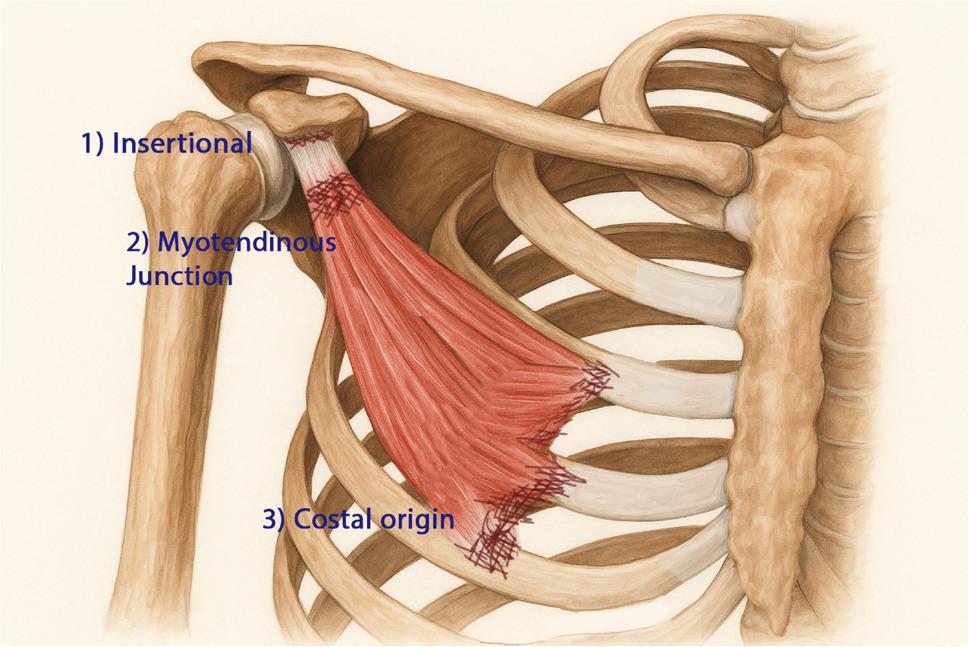
Schematic classification of isolated pectoralis minor tears

This tripartite classification provides a more comprehensive framework for understanding injury patterns and mechanisms and may facilitate future reporting consistency (Figure 3).

### Diagnostic implications

Because the PMi lies deep to the pectoralis major, tears rarely produce visible deformity. Pain is typically localized to the anterior shoulder or upper chest and may be misattributed to costochondritis, pectoralis major strain, or intercostal pathology [2, 3, 12]. In the present case, maximal tenderness over the third to fifth ribs near the costochondral junction suggested a more inferior lesion than those reported previously.

Magnetic resonance imaging remains the diagnostic modality of choice, allowing precise localization of the tear and reliable exclusion of concomitant pectoralis major or rotator cuff pathology [1–3, 6]. Recognition of associated rib bone marrow edema, as observed in this case, may further raise suspicion for a costal origin injury. Although rare, clinicians should also remain alert to potential neurovascular complications, as subclavian vein thrombosis has been reported in association with PMi tear.

Ultrasonography may allow dynamic assessment of superficial soft tissue injuries and has been reported as useful in selected pectoralis major pathologies. However, evaluation of the pectoralis minor muscle is technically challenging due to its deep anatomical location beneath the pectoralis major and proximity to the thoracic wall, making sonographic visualization highly operator dependent. In contrast, magnetic resonance imaging provides superior soft tissue contrast and reliable delineation of tear location, extent, and associated findings, such as adjacent rib bone marrow edema, as demonstrated in the present case. For these reasons, MRI remains the preferred imaging modality for suspected isolated pectoralis minor tears.

### Management and prognosis

Across all previously reported cases, management of isolated PMi tears has been uniformly nonoperative, consisting of rest, activity modification, physiotherapy, and gradual return to activity. Even professional athletes achieved full return to sport within weeks, and no patient required surgical intervention.

Although no standardized rehabilitation protocol has been established for isolated PMi tears, prior reports consistently describe conservative treatment with early pain-limited range of motion and progressive return to function. The phased rehabilitation approach used in the present case was intentionally conservative, emphasizing activity modification, avoidance of early resisted internal rotation or forceful scapular retraction, and delayed strengthening. This strategy aligns with the principles implicitly described in the existing literature while providing greater clinical detail to guide management.

The consistently favorable outcomes reported to date likely reflect the PMi’s secondary role in shoulder biomechanics, with limited contribution to power generation or joint stability [5]. The complete recovery observed in the present patient within six weeks further supports the effectiveness of conservative management across different tear locations.

### Clinical relevance

This case broadens the recognized anatomical spectrum of PMi tears to include the costal origin and highlights that such injuries may arise from low-energy, non-sporting mechanisms. Clinicians should consider this diagnosis in patients presenting with anterior chest wall pain, preserved pectoralis major contour, and focal costal tenderness. Early MRI evaluation can confirm the diagnosis, prevent unnecessary investigations or interventions, and facilitate appropriate conservative rehabilitation.

### Limitations

This report has several limitations. First, it describes a single patient, which limits the generalizability of the findings. Second, follow-up was limited to three months, and longer-term outcomes could not be assessed. Finally, although a detailed rehabilitation protocol is presented, no standardized rehabilitation guidelines exist for isolated pectoralis minor tears, and treatment strategies are largely based on limited case reports.

## Conclusion

Isolated tear of the pectoralis minor is a rare and frequently underrecognized injury that may mimic pectoralis major or costochondral pathology. Including the present report, twelve cases have been described in the literature, all of which were successfully managed without surgical intervention.

The present case describes a costal origin tear of the pectoralis minor associated with adjacent rib bone marrow edema following a low-energy, non-sporting mechanism, thereby expanding the recognized anatomical spectrum of this injury. Accurate diagnosis with magnetic resonance imaging is essential to avoid misdiagnosis and to exclude concomitant pathology. Based on available evidence, conservative rehabilitation remains an effective treatment approach, with favorable functional outcomes reported across different tear locations.

## Supplementary Information

Supplementary Material 1.

## Data Availability

All data generated or analyzed during this study are included in this published article. No additional datasets were generated.
