# Supplementary material for: Isolated pectoralis minor tear at the costal origin: a case-based review of the literature
Source: BMC Musculoskelet Disord. 2026 Jan 26;27:107. doi: 10.1186/s12891-025-09454-6 (PMC12879340; doi:10.1186/s12891-025-09454-6)
Supplement: Supplementary file 1 — Supplementary Material 1. [file 12891_2025_9454_MOESM1_ESM.pdf]

CARE
case report guidelines

CARE Checklist of information to include when writing a case report

(cc) BY-NC-ND

Topic	Item	Checklist item description	Reported on Line
Title	1	The diagnosis or intervention of primary focus followed by the words “case report”	Line 1-2
Key Words	2	2 to 5 key words that identify diagnoses or interventions in this case report, including "case report"	Line 62–63
Abstract (no references)	3a	Introduction: What is unique about this case and what does it add to the scientific literature?	Line 37–41
	3b	Main symptoms and/or important clinical findings	Line 42–44
	3c	The main diagnoses, therapeutic interventions, and outcomes	Line 44–49
	3d	Conclusion—What is the main “take-away” lesson(s) from this case?	Line 55–61
Introduction	4	One or two paragraphs summarizing why this case is unique (**may include references**)	Line 87–89
Patient Information	5a	De-identified patient specific information.	Line 91–93
	5b	Primary concerns and symptoms of the patient.	Line 93–96
	5c	Medical, family, and psycho-social history including relevant genetic information	Line 97–98
	5d	Relevant past interventions with outcomes	N/A
Clinical Findings	6	Describe significant physical examination (PE) and important clinical findings.	Line 102–115
Timeline	7	Historical and current information from this episode of care organized as a timeline	Line 91–115
Diagnostic Assessment	8a	Diagnostic testing (such as PE, laboratory testing, imaging, surveys).	Line 120–127
	8b	Diagnostic challenges (such as access to testing, financial, or cultural)	Line 227–246
	8c	Diagnosis (including other diagnoses considered)	Line 120–127
	8d	Prognosis (such as staging in oncology) where applicable	Line 147–151
Therapeutic Intervention	9a	Types of therapeutic intervention (such as pharmacologic, surgical, preventive, self-care)	Line 133–146
	9b	Administration of therapeutic intervention (such as dosage, strength, duration)	Line 133–146
	9c	Changes in therapeutic intervention (with rationale)	Line 133–146
Follow-up and Outcomes	10a	Clinician and patient-assessed outcomes (if available)	Line 149–151
	10b	Important follow-up diagnostic and other test results	N/A
	10c	Intervention adherence and tolerability (How was this assessed?)	Line 133–146
	10d	Adverse and unanticipated events	N/A
Discussion	11a	A scientific discussion of the strengths AND limitations associated with this case report	Line 265–278
	11b	Discussion of the relevant medical literature **with references**.	Line 188–264
	11c	The scientific rationale for any conclusions (including assessment of possible causes)	Line 280–290
	11d	The primary “take-away” lessons of this case report (without references) in a one paragraph conclusion	Line 284–290
Patient Perspective	12	The patient should share their perspective in one to two paragraphs on the treatment(s) they received	N/A
Informed Consent	13	Did the patient give informed consent? Please provide if requested	Yes ☒ No ☐
